# Time-Chunking and Hyper-Refocusing in a Digitally-Enabled Workplace: Six Forms of Knowledge Workers

**DOI:** 10.3389/fpsyg.2016.01627

**Published:** 2016-10-24

**Authors:** James E. Gaskin, Tanner Skousen

**Affiliations:** Information Systems, Brigham Young UniversityProvo, UT, USA

**Keywords:** digitalization, hyper-refocusing, time-chunking, task prioritization, time management strategies, new worker forms, task-switching, task-reconfiguration

## Abstract

Until the turn of the millennium, switching tasks required moving locations or reconfiguring physical workspaces. However, contemporary digital tools and workspaces allow knowledge workers to perform an increasingly diverse set of tasks, with an increasingly extending arm of influence, all from the same physical location without any physical reconfigurations or traversing of physical space. Along with this increased ambidexterity comes an increase in the quantity and frequency of demands on the time of knowledge workers. This digitalization of work now tethers their ability to perform largely to their ability to intensely focus in small chunks, and then “hyper-jump” that focus to another task without traversing the cognitive cool downs or warm ups required to reconfigure their train of thought from one task to another. Accordingly, they must become more like the hyper-functioning tools they employ if they are to avoid becoming the bottleneck resource in the configuration of socio-technical elements comprising their work routines. In order to better understand how knowledge workers manage their time and maintain focus across multiple and interrupting tasks, we leverage current time prioritization literature to propose and theorize around two key constructs: time-chunking and hyper-refocusing. By combining the possible values for these two constructs, we hypothesize the emergence of six forms of knowledge workers and their relative expected performance. The effects of digitalization on these new worker forms are discussed. We conclude by discussing opportunities for new research questions regarding time-chunking strategies and the hyper-refocusing ability.

## Introduction

Recently, technological progress in the form of digitalization—the infusion of digital capabilities into nearly all facets of work (Yoo et al., 2006)—has spawned new organizational forms and routines. Organizations are required to be more flexible, more fluid (Schreyögg and Sydow, 2010), and more “ambidextrous” (Raisch et al., 2009) in order to handle the increased complexity provoked by the digitalization of traditional work (Gaskin et al., 2014). Abundant research has documented the effect of digitalization on emergent organizational forms (Joyce et al., 1997; Robey and Jin, 2004; Gibson and Gibbs, 2006; Pentland et al., 2011). Perhaps less well-documented, however, are the effects of digitalization on emergent *worker* forms. Not only should the digitalization of work enable new forms of organizations, it should enable new forms of workers. Thus, scholars understand (or at least we have studied) to some extent how organizations need to adapt and react to the digital revolution in order to take advantage of digitalization. But we have not put the same energy into understanding how the foundation through which our organizations operate – i.e., the employees – might successfully leverage the effects of digitalization. It is imperative to better understand how individual employees can best leverage the effects of digitalization to accomplish their work, because gains in employee productivity (defined herein as value added/time invested) are gains to the bottom-line.

Exploring this issue requires careful consideration around the factors that may affect an individual’s response to digitalization. Many user behaviors and characteristics have been examined in theorizing whether a user will decide to use or continue to use a particular technology, but less has been done to examine the user strategies that affect effectiveness during use. Extant research on the effects of digitalization on work routines has explored, in particular, how interruptions due to technology affect worker focus (Rennecker and Godwin, 2005; Dennis et al., 2010) and task fragmentation (González and Mark, 2004; Mark et al., 2005). These studies tend to point out that digitalization has (1) increased the number of interruptions we receive, (2) fragmented our work into tiny chunks, and (3) compressed time and space so that workers are never unavailable—no matter the time of day/night or current location. The resulting implications offered by scholars tend to focus on redesigning technologies to better accommodate the increase in interruptions and fragmentation, emphasizing the need for more seamless and multi-tasking, synchronous and asynchronous communication technologies, and changes to organizational expectations and protocols to better accommodate these changes due to digitalization (Rennecker and Godwin, 2005; Mark et al., 2008; Duxbury and Smart, 2011).

The dominant approach has therefore been to try to shape the tools and environment around the worker in order to remove potential inhibitors of productivity. While this worthwhile effort may make great strides in opening the way for worker productivity, to some extent, it seems like patching^1^ the problem instead of redesigning for it. For now at least, the human worker is still the kernel^2^ resource in valuable knowledge work. Therefore, addressing the effects of digitalization by redesigning anything but the human knowledge worker^3^ will result in only incremental gains. In all fields, addressing the kernel (sometimes called the core) will always produce greater effects than addressing the periphery, because the kernel holds the greatest leverage. Accordingly, research on the effects of digitalization may profit by turning now to what the individual knowledge worker can do to adapt to the changes in his/her work environment due to digitalization.

In this article, we refer specifically to knowledge workers. Therefore, any reference to “workers” can be assumed to be knowledge workers. Along with defining the type of worker we are discussing, it is essential that we define our understanding of a task. A performed task could be comprised of many other elementary components, each of which is also a part of numerous other tasks (Gopher et al., 2000). For purposes of this paper we consider a task to be a goal-oriented activity that contains both a start and end (i.e., when the goal is achieved). Thus, when we refer to tasks, these are the tasks that a knowledge worker encounters, such as writing, researching, corresponding, developing, etc. With that clarification, we propose just one of many possible avenues to pursue as we study how to train knowledge workers to better adapt to the changing nature of work. Namely, we recommend exploring strategies for workers’ task prioritization and time management, and the individual and innate ability to focus intensely and continuously while addressing interruptions. This study seeks to extend existing research (such as Rennecker and Godwin, 2005; Reddy et al., 2006; Mark et al., 2008; Karagiannis and Vojnovic, 2009) by theorizing around refocusing abilities and time management strategies, and their possible effect on productivity. The primary research questions driving this study are: (1) How might workers with different refocusing abilities and time management strategies (and competencies) handle technology-induced interruptions? (2) How might the digitalization of work and tools help and/or hinder workers with different refocusing abilities and time management strategies?

To pursue these research questions, we bring together research on focus, interruptions, rhythms, prioritization strategies, and task reconfiguring. Using these streams of research as a guide, we further develop and explain two constructs: hyper-refocusing and time-chunking, to better understand and measure refocusing and time management strategies. These two key constructs are not entirely new. Hyper-refocusing is often referenced as task switching in cognitive psychology, and has been developed as an experimental paradigm to explore the mechanisms of cognitive control (Kiesel et al., 2010). Time chunking has been discussed in business and blogs, but has yet to be developed academically. We therefore extend the work around these two constructs and offer some initial theorizing around them. In doing so, we develop a framework for understanding the different strategies workers may employ for organizing time and tasks. This manuscript extends exploration and theorizing around these issues and opens new pathways to pursue. Accordingly, we conclude this article with new research questions, strategies for the measurement of the new constructs, and recommendations for future research.

### Guiding Literature

In this section we review the literature requisite for addressing the research questions. Specifically, we review studies on the following topics: (1) focus, (2) interruptions, (3) prioritization strategies, and (4) task reconfiguration. This literature review is not exhaustive, but focused on a handful of key articles that help illuminate the necessary paths to understand and develop the foundational constructs in this study.

#### Focus

Focus is the act of directing attention to a source of stimuli (Ocasio, 2011). When we focus intensely, we ignore external stimuli (such as interruptions) to our best ability, and we devote our cognitive resources to a limited set of stimuli (Lansman et al., 1983). Research on focus has a lengthy tradition, although focus is a crucial component of many newer theories (e.g., Agarwal and Karahanna, 2000). One way scholars have applied the concept of focus has been to explain *flow* (Voiskounsky and Smyslova, 2003; Sherry, 2004; Nakamura and Csikszentmihalyi, 2009). For example, Agarwal and Karahanna (2000) developed a construct called cognitive absorption which includes *focused immersion*, which is “the experience of total engagement where other attentional demands are, in essence, ignored.” Thus, as digital technologies have increasingly mediated and enabled our work, focus has become a vital construct in studies of human computer interaction (O’Conaill and Frohlich, 1995; McFarlane and Latorella, 2002; González and Mark, 2004; Oulasvirta and Salovaara, 2004; Mark et al., 2005, 2008).

The splitting of focus is of particular interest to the current study. Digitalization of work and tools has increased the number of “threads”^4^ we can manage seemingly simultaneously; thus, our focus is potentially fragmented in order to accommodate multiple threads. Along these lines, Lansman et al. (1983) conducted an experiment and reviewed the literature on focus to better understand “split-focus.” Their findings (as well as the literature they reviewed) provide consensus that a general ability to split one’s focus is not inherent in humankind. Instead, humans focus serially in order to attend to multiple competing stimuli. In their study of 85 subjects, Lansman et al. (1983) found that the best predictor of successfully accomplishing a “split-focus” task was actually the individual’s ability to accomplish single-focus tasks. Thus, even if we are handling multi-threaded work, we focus on only one thread at a time. Interestingly, this is also consistent with contemporary computer processors which can handle only one thread at a time, although they switch their focus so fast that they appear to be handling multiple threads simultaneously.

#### Interruptions

An interruption is a “synchronous interaction which is not initiated by the recipient, is unscheduled, and results in the recipient discontinuing their current activity” (O’Conaill and Frohlich, 1995, p. 262). The effects of interruptions on human cognition are fairly well-documented (e.g., O’Conaill and Frohlich, 1995; McFarlane and Latorella, 2002; Rennecker and Godwin, 2005). Although, some research has suggested that interruptions may have some positive effects (Gillie and Broadbent, 1989; O’Conaill and Frohlich, 1995; Jett and George, 2003), by and large, findings suggests interruptions lead to delays, errors, mistakes, and frustration (Oulasvirta and Salovaara, 2004; Mark et al., 2008). These negative effects are especially pronounced when the task being interrupted is one requiring heavier cognitive resources – such as when performing unstructured or novel problem solving (McFarlane and Latorella, 2002).

Perhaps the most thoughtful essay on digitally mediated interruptions is by Rennecker and Godwin (2005). In their study, Rennecker and Godwin (2005) explore the relationships between synchronicity of communication, interruptions and delays, and desired locus of control (among other constructs). They propose that the way workers manage interruptions is largely based on their desire for control. Workers who strongly desire to be in control often interrupt others using synchronous technologies (such as phone or instant messaging) in order to reduce the delay they might experience waiting for a response. However, they respond to interruptions asynchronously, if possible, in order to avoid losing their train of thought. Employees with less need for control are just the opposite—they interrupt others asynchronously and respond to interruptions synchronously. These behaviors are also affected by constructs such as power relationships, relationship affinity, and organizational culture.

#### Prioritization Strategies

A prioritization strategy is a set of heuristics guiding the position placement of tasks into our “queue” of things to do. According to Williams (2006), the innate human strategy for prioritizing tasks is driven by heuristics to minimize danger and maximize pleasure. Research has shown that, at the subconscious level, humans always have a primary task (i.e., the thing that is currently occupying their focus) and that all other tasks are interruptions that compete for cognitive resources (i.e., our focus) (Edwards and Rothwell, 2011). Whenever those cognitive resources are split among, or transferred between, multiple tasks, the performance of both tasks becomes poorer (Yogev-Seligmann et al., 2012). Accordingly, if allocating attention to the secondary task poses a threat to the primary task, often we will avoid the secondary task until the primary task can be paused without posing a threat. For example, if our primary task is driving and our secondary task is responding to a text message, we will (hopefully) delay allocating attention to responding to the text until such an action does not pose a threat (e.g., we arrive at a stop light or at our destination).

If a task is novel to us, it requires more cognitive resources and interruptions pose more serious threats (Edwards and Rothwell, 2011). When the interruptions are also novel, the threat is heightened (Bherer et al., 2005). For example, soon after one of the authors received his driver’s license he also received his first cell phone. While in traffic, his cell phone rang (something to which he was definitely not accustomed). In reaction to this novel interruption, he pulled the parking brake while merging onto the freeway… Needless to say, his performance definitely dropped for both tasks. On the opposite side of the spectrum, if an individual is experienced at each of the competing tasks, the tasks can often be done in parallel without large cognitive expenses or performance costs (Edwards and Rothwell, 2011). Consider the skilled parent who can cook while talking on the phone with a friend – all while soothing a distraught child. A less experienced parent will have to put down the phone and stop preparing the meal while soothing the child. Thus, for a less experienced individual, threat evaluation (meal burning, friend offended, child screaming) is an essential part of task prioritization.

#### Rhythms

Work rhythms describe the cyclical and repetitious nature of work for each worker. We have included rhythms as part of the guiding literature because constant repetition of a task enables a person through experience to become organized and to prioritize more efficiently (Feldman and Pentland, 2003; Becker et al., 2005). The cyclical nature of work was initially studied in the context of health workers to understand the temporality of work (Zerubavel, 1979). This classic study was further analyzed in order to discover how these rhythms affected group collaboration among hospital workers and how it affected the prioritization of tasks (Reddy et al., 2006). Reddy et al. (2006) discuss specific examples of how these rhythms affect prioritization of tasks. For example, consider the work loads of night shift nurses vs. day shift nurses. Both job functions are different, therefore if you asked a specific nurse to switch to the opposite shift, they would not have the experience or knowledge to effectively prioritize work tasks they will need to accomplish. Because of rhythms of work, Reddy et al. (2006) theorized that the experience and familiarity with certain tasks enhances the ability to prioritize tasks in the future.

While the study of rhythms is foundational to understanding that prioritization may be enhanced by the cyclical nature of work, our intention with hyper-refocusing and time-chunking differs from work rhythms, as we theorize the specific strategy of prioritization these workers employ either consciously or subconsciously.

#### Task Reconfiguration

Task reconfiguration refers to the rearranging of physical or cognitive resources in order to accommodate the switch from one focal demand to another. These types of switches happen with surprising frequency—on average, every 2–3 min for those who use both digital and analog technologies (González and Mark, 2004). Research on multi-tasking and task switching concludes unanimously that task reconfiguration is a time sink (e.g., Rogers and Monsell, 1995; Meiran, 1996; Monsell, 2003) – although it is common for workers to over-compensate for the lost time (by working faster), which has been shown to decrease total task completion time (Mark et al., 2008), albeit while increasing stress. Interruptions inevitably provoke task reconfigurations. Unfortunately, most research on task reconfiguration costs utilize basic abstract experiments involving overly simplified tasks, such as switching between adding and then subtracting single digit numbers (e.g., Rogers and Monsell, 1995; Meiran, 1996; Monsell, 2003) and, thus, cannot inform us about the costs associated with switching between real tasks—such as switching back and forth between writing a report and answering emails.

Nevertheless, given what we do know from these experiments on task switching, we may suppose that, at the very least, reconfiguration costs do exist in the form of time expenditure and cognitive reconfiguration for any type of task, however, large (Mark et al., 2008) or small (Monsell, 2003). Additionally, pausing after one task to gather thoughts and allow for “cognitive cool down” reduces ramp-up time and errors for the subsequent task (Rogers and Monsell, 1995). Lastly, multiple studies over a century of research have demonstrated the counterintuitive finding that reconfiguration costs are actually reduced when switching between two very different types of tasks and increased when switching between two slightly similar tasks (e.g., Jersild, 1927; Allport et al., 1994).

#### Time-Chunking

Time-chunking refers to allocating temporal blocks to specific tasks. For example, if you have a task that you estimate will require 3 h of your time, how do you allocate chunks of time for completing that task? Three seemingly mutually exclusive options^5^ are available to you: (1) block out the full 3 h by pushing other demands aside until the primary task is accomplished, (2) intentionally fragment the 3 h (or the task) into more manageable (but still rigid) chunks, or (3) allow the task to be elastic—to expand and contract, pause and recommence depending on context and environment (e.g., interruptions, priority changes). For convenience, we can refer to these as “blocked,” “fragmented,” or “elastic” time-chunking strategies^6^.

The time-chunking strategy we employ by default (and perhaps not intentionally or consciously) may depend on our ability to focus. If we know that we struggle to focus for long periods of time, perhaps a blocked approach wouldn’t be very effective, but a fragmented approach may work fine. If we are easily distracted, perhaps an elastic approach would not be very effective because it would not include definite time-based deliverables or fixed sub-tasks. If we struggle with reconfiguration costs (i.e., it takes us a long time to switch tasks), then we may prefer the blocked approach. Similarly, if a task has high priority—perhaps because not doing it endangers us (i.e., more pressing deadlines exist), or because doing it is pleasurable (Williams, 2006)—then we are likely to benefit more from a blocked approach. However, if we are anticipating several interruptions (perhaps because we work in a high-traffic area, or cannot isolate ourselves virtually), then we may need to use a more elastic approach. These are just a few of the factors that may affect the preferability, or the likelihood of employing, of each strategy. We found discussion of chunking in business blogs, yet no systematic study on the effects of chunking were identified. One blog states that chunking out tasks is always the best solution (Reh, 2016), but we hypothesize that the effectiveness of chunking may depend on various other factors^7^.

In **Table 1**, we theorize more systematically about these influential factors by breaking them up into task-related, personal, and contextual factors. **Task** factors refer to characteristics of the task to be accomplished. **Personal** factors refer to characteristics of the individual executing the task. **Context** factors refer to environmental characteristics external to the task and individual. Using these three factor types offers fairly broad coverage of potential influences without substantial overlap between factor types. In **Table 1**, we theorize around the *preferability* of each time-chunking strategy based on the various possible values for each of the factors listed under each of the three factor types. Thus, the values listed in a particular cell are preferable for the strategy listed in the corresponding row. For example, if a task is routine, then perhaps an elastic strategy would be most preferable because that task does not require the same amount of cognitive effort to switch between tasks. Whereas, a blocked approach would be an extremely monotonous strategy for the worker because of the routine nature of the task. Similarly, if a task is small, then perhaps a blocked strategy would be most preferable because it would only require a very small chunk of time to accomplish the task. Whereas, a fragmented strategy would be less preferable for small tasks because the ratio of reconfiguration costs to time required to accomplish the task would be inordinately high (because the task is so small). The values listed in **Table 1** are only a sampling of potentially influential factors rather than an exhaustive list. We expect there are many other factors in each category that may affect the preferability of a time-chunking strategy.

**Table 1:** A sampling of factors affecting the preferability of each time-chunking strategy.

Strategy	Influential factors		
	Task	Personal	Context
Blocked	–Medium or small size –Complex task –High priority –Novel task	–Can focus for extended periods –Gets distracted easily –Enthusiastic about task –High reconfiguration costs	–Sparse queue –Expecting few interruptions –Isolated physical work space –Virtual isolation is easy
Fragmented	–Large size –Complex task –Medium priority –Routine task	–Can’t focus for extended periods –Can ignore distractions with effort –Moderately enthusiastic about task –Low reconfiguration costs	–Full queue –Expecting some interruptions –Trafficked physical workspace –Virtual isolation possible in chunks
Elastic	–Medium or large size –Simple task –Low priority –Routine task	–Can focus in short bursts –Can ignore distractions easily –Less enthusiastic about task –Little to no reconfiguration costs	–Constantly changing queue –Expecting many interruptions –Shared or high traffic physical workspace –Virtual isolation is not possible

#### Hyper-Refocusing

The prefix “hyper” is used in conjunction with many words to denote ‘standing outside space or time.’ For example, hyper-jumping is moving instantaneously from Point A to Point B without traversing the intervening space. Hyper-text and hyper-media refer to a similar phenomenon in virtual space. These two examples of stepping outside of space restrictions also imply being outside of time, as the focal point can travel a distance in zero time. Therefore, there is no acceleration or deceleration when acting in a hyper-mode. Velocity is essentially constant.

Hyper-refocusing refers to the ability to task-switch without incurring substantive reconfiguration costs – i.e., focus is never “lost”; it simply jumps from one focal point to another. Someone who is able to hyper-refocus would, therefore, not suffer from slow cognitive deceleration and then acceleration when moving from a primary task to a secondary task. State of the art consumer computer products can already essentially do this, but can a knowledge worker do the same, or at least approach hyper-refocusing capability? When we pause writing a report to reply to an email, how long does it take us to accelerate into an effective “email writing velocity,” then decelerate once finished, and then accelerate back again into an effective “report writing velocity”? The same questions can be applied to any sort of task and interruption (e.g., driving and replying to a text).

The ideal, but likely unachievable, reconfiguration cost is zero—zero time required to reconfigure space and cognition to effectively begin executing (focusing on) the secondary task. Such ability would greatly enhance the productivity of one’s time. One who is able to hyper-refocus could be described as working outside the constraints of space or time as a fourth-dimensional worker.^8^ But does such ability exist? It is unlikely in the perfect form. Extant research cannot inform us precisely, as those studies have been abstracted away from real life tasks and only measure reconfiguration costs in terms of milliseconds between nominal tasks (e.g., Rogers and Monsell, 1995; Monsell, 2003). However, though we may not find any knowledge workers with the ability to hyper-refocus perfectly, these studies do reveal that the ability to refocus after task-switching does exhibit moderate variance across individuals (Lansman et al., 1983). Because of this variance of ability, we might apply deeper studies to the extremes on both ends of the spectrum in order to discover potential antecedents of the *near*-hyper-refocusing ability. Some studies have actually removed participant outliers from continuing with the experiments in order to study more consistent and generalizable behaviors and aptitudes (e.g., Lansman et al., 1983). However, it is these outliers that can better inform us regarding the ability or inability to approach hyper-refocusing.

### Theorizing New Forms of Workers

Is one time-chunking strategy more effective (produces better results) than another? Is hyper-refocusing generally more effective than not hyper-refocusing? Without thoughtful data collection, it is difficult to guess—although, hyper-refocusing surely has at least a time advantage due to reduced reconfiguration costs. However, saving time does not always mean producing better results. The more probable situation may be that none has an innate general advantage. Instead, it is likely their various combinations determine the context-dependent effectiveness. Thus, for one worker, a blocking strategy might be most effective in most cases; whereas for another, an elastic strategy would fit better with his/her ability to hyper-refocus. What would help then is a systematic treatment of the possible combinations of hyper-refocusing ability and time-chunking strategies. Such an approach would provide insight into the potential new forms of knowledge workers. In **Table 2**, we offer a 2x3 matrix of these new forms of knowledge workers as well as informal logic-driven hypotheses as to the expected *relative* productivity fit of each combination. *Relative* is a keyword here. Although, the ability to hyper-refocus may enhance any selected time-chunking strategy, we hypothesize that the strategy with the *best* fit for that individual would be elastic and the strategy with the worst fit would be blocked. This *does not* mean that someone who is skilled at hyper-refocusing will be less productive while blocking than someone who is unskilled at hyper-refocusing. **Table 2** also theorizes without consideration of task or context related factors.

**Table 2:** Relative productivity fit for different forms of knowledge workers.

Time-chunking strategy	Hyper-refocusing ability	
	Unskilled	Skilled
Blocked	Best fit	Poor fit
Fragmented	Moderate fit	Moderate fit
Elastic	Poor fit	Best fit

Each of the combinations of time-chunking and hyper-refocusing should have a relative fit with each other and a resultant correlation with productivity. For example, if a worker is unskilled at hyper-refocusing, the best time-chunking strategy for maximizing productivity should be a blocked strategy because such a strategy minimizes reconfiguration costs by reducing the frequency of task-switching. Conversely, an elastic strategy would be a very poor fit because it increases task-switching frequency and would therefore lead to time loss due to excessive reconfigurations. For a worker who is more skilled at hyper-refocusing, the opposite combinations and results are hypothesized: blocked is a poor fit and elastic is a good fit. Someone who is skilled at hyper-refocusing should be able to accomplish more by treating interruptions synchronously (i.e., elastic time-chunking), as this will keep his/her queue uncluttered and will clear out request inventory. Lastly, a fragmented strategy is likely to fit somewhere in between because it allows for predictable fragmentation of tasks into a few rigid blocks of time. Thus, someone who is unskilled at hyper-refocusing will benefit by having a limited and predictable amount of task-switching (less than an elastic approach), but will be hindered by the additional reconfigurations (more than the blocked approach). One who is skilled at hyper-refocusing will benefit by the opportunity to address other tasks in between time chunks (although less than the elastic approach), but will be hindered because of the rigid constraints within those chunks (although more flexible than the blocked approach).

### The Effects of Digitalization

Not too many years ago, digital technologies touched the work of individual knowledge workers far less. All else equal, tasks of stark variety presented themselves less frequently back then than in our current workplace. Consider just 20 years ago the lawyer who, preparing for a case, compiled support into a portfolio of evidence. The lawyer would block out several hours or a day, or even a few hours a day for a week or two to spend in the library. While there, few if any interruptions occurred. Mail in the inbox was truly asynchronous (and physical), and therefore could not interrupt the researcher while in the library. Compare this to the current scenario where the task of compiling evidence for a portfolio is done using the same physical configuration (laptop, office, desk, chair) as many other tasks (including receiving and responding to mail). Now only the virtual configuration must alter to attend to a different task—and these virtual configurations are shrinking as the affordances of individual digital technologies expand and become hyper-multi-functioning. There is no end to technologies that are affecting our work with technologies such as email, the internet, databases, phones, tablets, and more. This story of times changing due to digitalization is well-known and well-documented (e.g., Davis, 2002; Rennecker and Godwin, 2005). Novel to this study, however, are the effects of digitalization on different forms of workers. We next discuss how each of the three proposed time-chunking strategies is affected by digitalization, as well as the effects of digitalization on the relative ability to hyper-refocus.

#### Digitalization’s Effect on Blocked Time-Chunking

Blocking large chunks of time may actually be more possible now than before because we can virtually (i.e., digitally) isolate ourselves if necessary by turning off our email client, silencing our phone, etc. However, blocking is more hazardous now because the increase in secondary tasks, due to digitalization, creates task stock piles when we ignore them (e.g., our inbox fills up). Additionally, those doing the interrupting know that we are accessible at any time (Duxbury and Smart, 2011) – and often they are aware if we have received or viewed their request on our time. Thus, pushing back their requests until you complete your block of time may risk relationships (Rennecker and Godwin, 2005). Digitalization may have also turned blocked time chunking into an escape mechanism rather than a *modus operandi*. When the influx of secondary tasks becomes overwhelming and we find we are slipping behind on our primary tasks, we may resort to a blocked strategy in order to accomplish more pressing, high priority tasks.

#### Digitalization’s Effect on Fragmented Time-Chunking

Fragmenting our work is much less costly than it used to be. Consider a familiar example; a researcher (a type of knowledge worker) conducting a literature review. Fragmenting the literature review 20 years ago meant traveling back and forth from the library for each fragmentation. Now, the only space the researcher must traverse is virtual (open browser, start search) and takes fractions of seconds rather than fractions of hours. Fragmentation is also bolstered by digitalization because digitalization has increased the frequency of requests on our time by others for secondary tasks (Davis, 2002). Thus, we have lots of little pebbles or rock fragments to fit into the figurative time jar rather than just a few large rocks. Therefore, we can fill the time between our fragmented time-chunks with many other small secondary tasks; thus reducing the effect of delays previously prone to fragmented or interdependent work (Rennecker and Godwin, 2005).

#### Digitalization’s Effect on Elastic Time-Chunking

Perhaps elastic time-chunking was born out of digitalization—although, one can imagine the scenario where a person in high-demand (e.g., CEO, President) *could have* worked elastically in the past *if* all those with demands flocked around him/her whenever they demanded his/her attention. Regardless, digitalization has certainly enabled more workers to chunk their time elastically than ever before. For example, as one of the coauthors, as I sit here writing this manuscript, I have answered email requests and forum inquiries from perfect strangers across the globe, given feedback on other manuscripts I’m coauthoring, attended to several knocks at my door, updated software on my laptop, and replied to a text from a colleague. My reaction to most of these secondary tasks has been fairly synchronous and yet the momentum of this manuscript has hardly waned because digitalization of tools and tasks increases the elasticity potential of my focus and my tasks.

This increase in elastic potential may be largely due to the ‘delayed-synchronous’ potential of digital interruptions. Since most digital requests are made remotely, they can be addressed with a delayed-synchronous approach. This approach enables the worker to acknowledge an interruption (e.g., email), but wait to address it for another 30 s or so until the primary task is at a good break point.^9^ To the one making the interruption, the response is still sufficiently synchronous. Thus we are warping perceived (present) time by maintaining perceived synchronicity even while delaying the response. This “time warping” made possible by digitalization is a tell-tale characteristic of an elastic time-chunking approach.

#### Digitalization’s Effect on Those Who Hyper-Refocus Poorly

For the worker who struggle with task transitions, digitalization is a boon because it retains the exact state of our work intact between interruptions. Thus, when we are interrupted, or must fragment our work, the reconfiguration cost of returning to the primary task is decreased. Digitalization also enables such workers to isolate themselves from interruptions (turn off email client, silence phone, etc.) more effectively than in the past when most interruptions came calling at our physical doors. Thus, for those who do not hyper-refocus very well, digitalization can act as a shield against costly task-switching due to interruptions. However, one potential drawback of digitalization is that when we virtually isolate ourselves, the ignored interruptions leave residue tasks in a pile waiting for our return from isolation (although some workers certainly opt to ignore the interruptions even then – which explains the great phenomena of unreplied emails…). Whereas, if you don’t answer your physical door, the interruption might just go away. Lastly, if those unskilled at hyper-refocusing do not consciously apply the strategies mentioned above that beneficially leverage digitalization, they risk falling into an incompatible strategy (such as an elastic strategy) as they try to react to the sheer amount of interruptions prone to this digital age.

#### Digitalization’s Effect on Those Who Hyper-Refocus Well

Digitalization is also a boon for the skilled hyper-refocuser because it allows him/her to address all tasks and interruptions using a live queue (i.e., interruptions can be addressed relatively synchronously). Thus, backlogs of secondary tasks are less likely build up. However, a skilled hyper-refocuser may also suffer from the effects of digitalization if the interruptions never cease (this may happen when responding to one interruption prompts a follow up interruption)—effectively putting the worker’s primary task on permanent hiatus. Accordingly, task prioritization becomes a prominent concern due to digitalization. If we have more interruptions than we can handle synchronously, which ones do we handle now and which ones do we place later down in the queue? The mindful worker will likely follow the threat/pleasure model discussed earlier (Williams, 2006).

## Discussion and Future Directions

This study has been guided by two main research questions: (1) How might workers with different refocusing abilities and time management strategies (and competencies) handle technology-induced interruptions? (2) How might the digitalization of work and tools help and/or hinder workers with different refocusing abilities and time management strategies? Guided by these two research questions, we have reviewed the literature on (1) focus, (2) interruptions, (3) prioritization strategies, and (4) task reconfiguration in order to develop and extend two key constructs related to time management: (1) time-chunking and (2) hyper-refocusing. Drawing upon the reviewed literature, we have theorized around new forms of workers by combining the different values of time-chunking and hyper-refocusing. The theorizing and informal hypotheses guess at the relative effectiveness of each combination (i.e., each form knowledge worker). This exploratory theorizing is followed up with an examination of how each time-chunking strategy and each hyper-refocusing ability is affected (for good or ill) by the digitalization of work and tools. In direct response to our research questions, **Table 2** addresses our first research question, while our second research question is addressed in the section on the effects of digitalization, which effects are partially summarized in the bullet points below:

• Digitalization has increased interruption frequency, but digitalization has increased our control over interruptions (and when we respond to them).
• Digitalization makes blocked time-chunking easier, but more hazardous.
• Digitalization makes fragmented time-chunking less costly and more productive.
• Digitalization makes elastic time-chunking available to more types of workers.
• Digitalization decreases reconfiguration costs for those unskilled at hyper-refocusing.
• Digitalization enables those skilled at hyper-refocusing to process their queue of tasks synchronously; however, digitalization potentially creates an endless queue if prioritization is neglected.

Some important questions which remain to be explored may guide future research on this topic. We list four primary sets of questions here, after which we discuss them and propose additional secondary follow-up questions.

(1) To what extent can individuals actually hyper-refocus? What is the distribution of hyper-refocusing ability among a sample of “typical” knowledge workers and how can we measure it? Does this distribution vary depending on contextual, individual, or task-based factors?
(2) To what extent are the three time-chunking strategies proposed in this essay utilized by “typical” knowledge workers and how can we measure them? Are these strategies consciously, or subconsciously applied? What factors influence the adoption of these strategies? Are these strategies constant for individuals or are they context-dependent?
(3) To what extent does the proposed productivity-fit across combinations of time-chunking and hyper-refocusing (**Table 2**) actually match data-driven evidence? Do workers tend to create an affinity for one time-chunking strategy based on their hyper-refocusing ability? For example, do skilled hyper-refocusers tend to adopt an elastic strategy?
(4) To what extent can the ability to hyper-refocus be acquired over time through practice or training? Or is hyper-refocusing an inborn, relatively constant trait akin to intelligence? If learnable, how – through what specific training or practice?

Regarding the training/learning of hyper-refocusing, we posit that hyper-refocusing probably behaves a lot like one’s intelligence quotient (IQ). To be clear, we are not suggesting IQ and the ability to hyper-refocus are positively correlated. We are simply arguing that the ability to hyper-refocus is likely as constant as IQ, which has a sort of genetic base-level that varies across individuals, but it can increase or decrease over time depending on the conditions of our experiences (Loehlin et al., 1989). However, just as all changes in IQ are anchored to that genetic base-level, so might be one’s hyper-refocusing ability. As for the *application* of hyper-refocusing, it probably also follows the application of IQ. That is to say, co-present contextual factors such as distractions, mood, health, etc. can significantly affect application (Moser et al., 2011).

As for time-chunking, we posit that individuals likely do not employ only one strategy. Instead, they might employ each time-chunking strategy based on the factors, among others, listed in **Table 1**. Nevertheless, they may have an affinity for one strategy above another. As one of the coauthors, my personal default is to take an elastic approach to time-chunking. However, I sometimes will use a fragmented approach when it suits the task and my current set of demands better. On very rare occasions I use a blocked approach—usually when a task has been pushed down my queue so often and for so long that it is reaching the point of criminal neglect. Variance most certainly exists among a larger sample of knowledge workers. Another question to pursue, in this regard, is whether workers consciously adopt particular time-chunking strategies, or if they employ them subconsciously. If subconscious, can they identify the strategy if prompted? In addition, do the factors listed in **Table 1** sufficiently capture the factors that may affect the preferability of time-chunking strategies? Which are most influential? Are there other influential factors?

Additionally, assuming the six forms of knowledge workers proposed in **Table 2** can actually be found in modern workers, do those forms correlate with different types of outcomes? For example, do workers in the bottom right of **Table 2** (hyper-refocusers, elastic time) tend to produce a greater number of contributions but with shallower impact? Do the workers in the top left of **Table 2** (non-hyper-refocuser, blocked time) tend to produce fewer, but deeper contributions with greater impact? If so, we may have found the “movers” (who get things done) and “shakers” (who change the landscape) of whom O’Shaughnessy (1874) wrote. But these are just postulations.

Because this theory has yet to be empirically validated, we hesitate to offer any form of practical implications. At this early stage of exploratory theorizing, the logical next step is to seek to empirically validate and refine the ideas put forth in this manuscript. Our recommendation for progressing this work is to engage first in qualitative interviews and observations in order to determine if time-chunking and hyper-refocusing are actually occurring and if there seems to be non-random variance. Interviews may also reveal when, how, and under what circumstances hyper-refocusing and each time-chunking strategy are employed. Such an approach may also help refine the three proposed time-chunking strategies and may also reveal a more continuous spectrum of hyper-refocusing than the relatively binary one we have theorized around. See the appendix for our preliminary forays into measures and data collection strategies. Insights from these qualitative pursuits could then be applied to the development of appropriate measures and studies for capturing these constructs and their possible effects on outcome variables such as worker productivity, satisfaction, perceived collegiality, or effectiveness. To conclude this study, we next offer one set of possible strategies for measuring time-chunking and hyper-refocusing.

## Conclusion

Digitalization of work and tools is changing the way organizations must behave. As a result, we see new organizational forms emerging. In this essay we argue that individual workers must also adapt to leverage the effects of digitalization. In doing so, we may see new worker forms emerging. We propose six forms of knowledge workers characterized in terms of their strategy for time-chunking and their ability to hyper-refocus. Some of the effects digitalization has on these worker forms are identified; namely: digitalization makes blocked time-chunking easier but more hazardous, fragmented time-chunking less costly and more productive, and elastic time-chunking available to more types of workers. Digitalization also decreases reconfiguration costs for those unskilled at hyper-refocusing while enabling those more skilled at hyper-refocusing to process their queue of tasks relatively synchronously. Future research may want to focus efforts on better understanding the interaction of these two constructs, their distributions across typical knowledge workers, what affects them and what they affect. This might be achieved through both inductive qualitative research as well as more generalizable quantitative assessments of time-chunking strategies and their effects. We explore these opportunities in the appendix. The several proposed research questions will hopefully prompt future studies of time-chunking and hyper-refocusing.

## Author Contributions

JG wrote the original article and advised on the revision (previously rejected from ISR). TS conducted the bulk of the work for the major revision prior to submission to Frontiers. JG and TS collaborated on the first round revision at Frontiers.

## Conflict of Interest Statement

The authors declare that the research was conducted in the absence of any commercial or financial relationships that could be construed as a potential conflict of interest.

## Footnotes

1 In computer science, patching refers to creating workarounds (or patches) to help the original application be compatible with external changes that emerge over time.

2 In computer science, the kernel is the nucleus of a sophisticated application, like an operating system. The kernel manages all input and output requests.

3 We define knowledge workers as those whose primary responsibilities require handling, leveraging, or producing information.

4 In computer science, a thread refers to a basic task focused on by the processor. Current programming languages and processors accommodate “multi-threading” – seemingly (but not actually) processing multiple concurrent threads with a single processor.

5 A thorough search of the literature, using keywords like “time chunking,” “time blocking,” and “time fragmentation” strategies, returned no insights on options for time-chunking strategies. Thus, these three strategies are simply three logically conceivable options.

6 We offer a quick comparison here between time-chunking and working-spheres (González and Mark, 2004, p. 117). Time-chunking refers to the choice-driven strategy for fragmenting time or tasks into *temporal* chunks. Working-spheres refer to “a set of interrelated events, which share a common motive (or goal)” (González and Mark, 2004, p. 117). Thus, whereas time-chunking is about temporal allocations; working-spheres are more about task-type allocations.

7 Compartmentalization is one such factor that could affect the worker’s ability to effectively time-chunk. Compartmentalization, or the ability to divide tasks, behaviors, or aspects of one’s work or life into categories and subcategories, could be an enhancer of time-chunking strategies, or even a strategy in and of itself. While we do not theorize in this paper the implications of compartmentalization, we are aware of the possible connection between the two ideas. We argue that they are different, as time-chunking is about dividing your tasks into specific time-based portions, while compartmentalizing is typically about dividing various aspects of your life or work into roles or categories – irrespective of time.

8 The label is not perfect. A “fourth dimensional being” perceives time as we perceive space (all at once), and can move about in time as we move about in space (i.e., freely, c.f Abbott, 1885); whereas we have to wait for time to come to us. However, a fourth dimensional worker still can only work forward in time (cannot utilize negative time). Thus, the label “fourth dimensional worker” simply implies that the worker avoids the costs of time by not having to take the time to traverse the cognitive slopes of reconfiguring his/her focus.

9 We do not fully address “break points” in this manuscript; nevertheless, we define break-points here as a naturally occurring sub-termination point at which the cost of task-switching is minimized. The cost is minimized because the work leading up to this break point has sufficiently completed a modular subtask, and the worker has not yet begun on a new subtask.
