# Supplementary material for: Time-Chunking and Hyper-Refocusing in a Digitally-Enabled Workplace: Six Forms of Knowledge Workers
Source: Front Psychol. 2016 Oct 24;7:1627. doi: 10.3389/fpsyg.2016.01627 (PMC5076381; doi:10.3389/fpsyg.2016.01627)
Supplement: Supplementary file 1 [file Data_Sheet_1.docx]

# Appendix: Measurement strategy

We have recommended pursuing this area of research by first conducting qualitative research in order to explore more deeply the constructs of time-chunking and hyper-refocusing. The findings from the qualitative studies should then be integrated into follow-up quantitative studies to discover if these constructs can be measured reliably, and how they affect other variables and relationships. Accordingly, we next propose and describe a measurement strategy for both qualitative and quantitative approaches to study time-chunking and hyper-refocusing. We focus only on these two constructs (instead of branching out to theoretically related constructs such as reconfiguration and task switching; however the methods of measurement for some of these additional constructs are found here in the appendix), as these are the only novel constructs discussed in this essay, and because initial follow-up studies should focus on refining these constructs and finding better ways to validly measure them.

### Time chunking

There are certainly many methods for extracting this kind of information from workers, including basic observations/shadowing or semi-structured interviews. If we assume that time-chunking is an abstract concept that is not deliberately strategized by the typical knowledge worker, then we may have difficulty extracting reliable information about time-chunking strategies if we rely only on traditional approaches to qualitative inquiry. The worker simply might not know (or be able to articulate) how he/she typically chunks time. However, if we offer the worker a means of bringing to life or illustrating his/her thoughts, this will not only allow the worker to recognize the accuracy of his/her responses as they are given, it will also provide (for the researcher) a supplemental visual confirmation to the verbal responses. Optionally, this approach to semi-structured interviewing may benefit from video recording rather than just traditional audio. But how does one illustrate his/her thoughts? We offer one possible approach based on the qualitative questions listed below.

#### Qualitative Protocol

For this interview, the researcher will need a handful of coins of various denominations and the questions listed below.

1. The coins on the table each represent a task. The larger the denomination, the larger the task. In this case, pennies are the smallest types of tasks and quarters are the largest. Please use the coins on the table to illustrate your responses.
2. Please explain (and illustrate with the coins) how a typical workday develops in terms of task scheduling, processing, and prioritization. (It may be helpful to provide a very brief illustration of what you mean. For an example, “*I could have a pile of pennies representing small tasks that I get rid of at the beginning of my day because they are easy to finish before my first meeting. This results in the quarters (representing larger tasks) being pushed to the back of the queue*.” For further examples, watch this video: <http://www.youtube.com/watch?v=lnvXOFEfm6o>).
3. Please explain (and illustrate with the coins) how you handle interruptions throughout the day. (It might be necessary to dig deeper by asking about interruptions of different sizes and priority, or that occur early or late in the day.)
4. Please offer the same explanations but this time for a week.
5. Please offer the same explanations but this time for a month.
6. Please offer the same explanations but this time for the long-term (multiple months/years).
7. What sorts of factors affect your task prioritization?
8. What sorts of factors affect the way you block or schedule your time?

#### Quantitative Measures

From a more quantitative perspective, time-chunking strategy offers a clear moderating variable for regressions or a factoring variable for difference of means tests (such as ANOVAs). For example, we may want to determine the way each time-chunking strategy changes the relationship between one’s ability to hyper-refocus and his/her productivity. Or we may want to simply see if one time-chunking strategy is more productive than another strategy. The items we propose below are one way to determine with which time-chunking strategy a worker most associates. we use the ranking approach rather than a Likert-scale because, for a multi-group moderator or factoring variable, we only need to know group membership, rather than the extent to which one engages in each type of behavior. However, if one wanted to measure each time-chunking strategy as an ordinal latent variable, the items we list below may easily be adapted to a Likert-scale.

*Below are listed several sets of three behaviors. Within each set of three, please rank the behaviors in the order that best describes the way you work. The top item should best describe you.*

| Set 1 | Blocking1. I reserve large blocks of time to focus on a single task.  Fragment1. I reserve small blocks of time to focus on portions of a single task.  Elastic1. I do not reserve blocks of time to focus on a single task. |
| --- | --- |
| Set 2 | Blocking2. I do not address other tasks until I finish with the one I’m currently working on.  Fragment2. I do not address other tasks until I arrive at a good stopping point with the one I am currently working on.  Elastic2. I address tasks as they arrive, even if I’m working on something else. |
| Set 3 | Blocking3 I do not allow other tasks to interrupt my current task.  Fragment3 I allow interruptions only if I’m in between tasks.  Elastic3 I almost always allow interruptions at any time. |
| Set 4 | Blocking4 I ignore emails and other requests if I am in the middle of something else.  Fragment4 I ignore emails and other requests unless I can easily tie off loose ends for my current task.  Elastic4 I address emails and other requests even if I am in the middle of something else. |

*To score these measures, add the four rank values together for each type of strategy. The strategy with the lowest score is the dominant time-chunking strategy employed by that respondent. If two or more summed ranks are equal, then this could be considered a fourth “strategy” we could label as “no dominant strategy”.*

### Hyper-refocusing

As with time-chunking, hyper-refocusing may be an abstract enough construct that interviewed workers might need additional assistance in discovering and describing their own ability to hyper-refocus. Accordingly, we recommend augmenting semi-structured interviews with illustration objects—in this case, a whiteboard or paper and pencil. This will allow the worker to draw what they mean and then even revise it if the illustration is not what they intended. This allows the worker to discover while disclosing, and thus should provide a more accurate picture of their ability to hyper-refocus than if they simply described it orally. The interview protocol proposed below is just one approach for better understanding how and to what extent workers hyper-refocus.

#### Qualitative Protocol

For this interview, the researcher will need a whiteboard, or paper and pencil, and the questions listed below.

1. [Facilitator draws “A” and “B” a distance apart on the whiteboard, then hands the marker to the worker.]
2. Please explain and illustrate your level of focus as you move from Task A to Task B.
   1. Is this dependent on other factors?
3. When you move from Task A to Task B, how long does it take (in relative terms) to get into an effective groove for Task B?
4. [Facilitator draws a continuous line on the whiteboard.]
5. If this line represents your focus as you are processing your current task, describe and illustrate how interruptions during that task affect your level of focus. (It might be necessary to dig deeper by asking about interruptions of different sizes and priority, or that occur early or late in the day.)
   1. Is this dependent on other factors?

#### Quantitative Measures

From a quantitative perspective, we could collect an ordinal range of hyper-refocusing ability. To do this, we propose seven possible reflective items on a 5-point Likert-scale. These items could then be used to create an aggregate score for hyper-refocusing ability, or they might be used as a latent variable to predict other variables or be predicted by them. For example, we might test whether an increase in hyper-refocusing ability increases worker productivity.

*Please indicate the extent to which you agree with the following items as they describe you
(1 = Strongly Disagree; 5 = Strongly Agree).*

- HypRefoc_1 *Interruptions really derail my train of thought.
- HypRefoc_2 *Refocusing after switching tasks takes some time.
- HypRefoc_3 *I need some time to gather my thoughts before switching to a new task.
- HypRefoc_4 *Getting into a good working groove requires real effort on my part.
- HypRefoc_5 I can jump from task to task without much effort.
- HypRefoc_6 I’m up and running at full speed almost immediately after starting a new task.
- HypRefoc_7 Interruptions don’t affect my ability to focus on the task at hand.

**reversed*

### Measurement of Related Constructs

As mentioned in the article, task switching and task reconfiguration are related to hyper refocusing. For supportive information, below we provide the methods used to measure the constructs of task reconfiguration and task switching.

#### Task Reconfiguration

The following method was used by Gonzalez and Mark (2004) to study task reconfiguration.

*“The study was based on two main ethnographic techniques: participant observation and use of long interviews…In our case, the researcher sat with the informant at her cubicle and followed her, whenever possible, to meetings or other activities. The researcher sat just behind the informant where it was possible to fully observe what she did and to some extent to be able to read documents displayed on the computer screens, the ID caller display on the phone unit, the content of print outs, sticky notes and binders on the desk, etc. Whenever the individual performed an action such as opening a computer application, making a phone call, writing down a note on her planner or pulling a paper note from the cubicle wall, the researcher annotated the time (to the second) and other details of the event. All interactions with others were also documented, including details about the topic of the conversation, documents used and persons involved. We designed an activity tracking log where we transcribed the observation notes collected during the day. In the tracking log we included the time stamps, data about the type of event (e.g. “responding email to AMX”, or “modifying the Java code for CEW module), the resources used during the action (e.g. phone, Excel, planner, sticky note, calendar), and the people participating in the event. The tracking logs were used in the analysis. A total of 477 hours was spent in observation at the field site.”* (p. 114)

Meiran (1996) conducted an experiment using a 2x2 grid system. They termed this 2x2 grid as the cue. Their intent was to measure the between-task interval. “A session began with instructions that were presented on the monitor and followed immediately by 140 experimental trials. Each trial consisted of the cue, which was presented for either a short (203 ms) or a long (1,423 ms) cue-target interval, after which the target was presented along with the cue for an additional 1,500 ms. Participants responded by pressing keys on the keypad on a standard extended keyboard that was shifted to the left to align it with the screen. The intertrial interval was 1,138 ms, and during that time, the task, target, and cue-target interval were selected randomly with equal probabilities.” (p. 1428)

#### Task Switching

The following method was implemented by Monsell (2003) to measure task switching.

*“In a task-switching experiment, subjects are first pretrained on two or more simple tasks afforded by a set of stimuli. Each task requires attention to, and classification of, a different element or attribute of the stimulus, or retrieval from memory or computation of a different property of the stimulus. Then, a stimulus is presented on each of a series of trials and the subject performs one of the tasks. There are several methods for telling the subject which task to perform but in all cases the task sometimes changes from one trial to the next, and sometimes does not. Thus, we can examine performance or brain activation on and following trials when the task changes for evidence of extra processing demands that are associated with the need to reconfigure task-set. We can also examine the effects of localized brain damage, transient magnetic stimulation (TMS) or pharmacological interventions on behavioral indices of switching efficiency.”* (p. 135)
